# Mapping the evolution of botanical interventions for diabetic neuropathy: a two-database bibliometric landscape from 2005 to mid-2025

**DOI:** 10.3389/fnut.2025.1727582

**Published:** 2026-01-07

**Authors:** Xuankai Cui, Yilin Liu, Zhipeng Guo, Shuhan Yang, Jingni Wang, Yisong Liu, Xingchun Wang, Xinrui Wang, Xingxia Wang

**Affiliations:** 1 Department of Neurology, The Affiliated Hospital, Southwest Medical University, Luzhou, Sichuan, China; 2 Department of Clinical Medicine, School of Clinical Medicine, Southwest Medical University, Luzhou, Sichuan, China; 3 School of Information and Software Engineering, University of Electronic Science and Technology of China, Chengdu, China; 4 The Affiliated Stomatological Hospital, Southwest Medical University, Luzhou, Sichuan, China

**Keywords:** bibliometrics, botanical interventions, diabetic neuropathy, traditional Chinese medicine, visualization

## Abstract

**Objective:**

This study conducts a comprehensive bibliometric analysis to map the evolutionary trajectory, identify research hotspots, and forecast future trends in botanical interventions for diabetic neuropathy (DN) from 2005 to mid-2025.

**Methods:**

We retrieved relevant publications from the Web of Science Core Collection and PubMed (2005 to mid-2025). After applying inclusion criteria and removing duplicates, 414 articles and reviews were analyzed using VOSviewer, CiteSpace and Bibliometrix to visualize publication trends, collaboration networks, and keyword dynamics.

**Results:**

Annual publication output exhibited exponential growth after 2020, peaking at 71 publications in 2024. China dominated the research output, followed by India and the USA, though international collaboration remained limited. Keyword analysis identified four major research hotpots: molecular mechanisms (e.g., oxidative stress, NF-κB), clinical translation, systems pharmacology of traditional Chinese medicine (TCM), and signaling pathways. Burst detection revealed “network pharmacology” and “TCM” as the current research frontiers.

**Conclusion:**

Research on botanical interventions for DN is rapidly expanding, with a clear shift toward mechanistic and computational approaches. Future efforts should prioritize robust clinical trials, international cooperation, and deeper mechanistic studies to translate botanical potential into evidence-based therapies.

## 1 Introduction

According to the latest data from the 11th Edition of the *IDF Diabetes Atlas 2025* by the International Diabetes Federation (IDF), an estimated 588.7 million adults (aged 20–79) were living with diabetes in 2024, representing 11.1% of the world’s population in this age group. Moreover, diabetes prevalence increases with age, with adults aged 75–79 having a prevalence of 24.8%, far higher than that of adults aged 20–24, which is only 1.9%. Furthermore, considerable epidemiological variation exists between countries as well as between urban and rural areas. In 2024, the countries with the highest number of adults aged 20–79 living with diabetes were China, India, and the United States. However, globally, over four in ten (42.8%; 251.7 million) adults living with diabetes (20–79 years old) were undiagnosed. IDF projects that by 2050, driven by population aging and accelerated global urbanization, around 852.5 million individuals (13.0% of the global population in the 20–79 age group) will be affected by diabetes, marking a 45% rise from 2024^1^ (1, 2). In high-income countries, diabetes is the main cause of cardiovascular diseases, blindness, kidney failure, and lower limb amputations (3). Among the complications of diabetes, diabetic neuropathy (DN) is one of the most common and disabling complications, posing a serious challenge in global public health (4). This disease is not a single condition but consists of peripheral neuropathy, autonomic neuropathy, proximal neuropathy, and focal neuropathy. The core etiologies are all associated with long-term hyperglycemia, which damages nerves and their nutrient vessels, accompanied by metabolic factors such as dyslipidemia and hypertension. Among them, peripheral neuropathy is the most common (5, 6), characterized by symmetric sensory-motor nerve damage in the distal extremities, manifesting as pain, numbness, abnormal sensations (7), even foot ulcers and gangrene, which significantly impairs the quality of life of patients and greatly increases the risk of amputation and death (8), causing a heavy social and economic burden (9). Hyperglycemia, hypertension, hyperlipidemia, smoking, race, male gender, obesity, age, and genetic factors are the primary risk factors for the development and progression of DN. African Americans, Asians, and Native Americans have higher incidence rates of DN compared to Caucasians (10). Unfortunately, current pharmaceutical treatment strategies, such as pregabalin, duloxetine and 8% capsaicin patches (9), although able to alleviate pain symptoms to some extent, currently lack effective drugs for DN relief, and are often accompanied by significant adverse reactions. Non-invasive brain and nerve stimulation techniques have been proposed as potentially beneficial for DN, but their long-term efficacy has not been confirmed (11), and most of these techniques cannot cure DN, highlighting the substantial unmet need in clinical treatment (12, 13).

Against this backdrop, botanical interventions represent a promising area of investigation for the modulation of DN, largely due to their multi-target characteristics. The core logic lies in utilizing the multi-component characteristics of natural plants to achieve multi-target and holistic regulation of the body. In the context of botanical interventions, traditional Chinese medicine (TCM) is the primary intervention method due to its long history of clinical application and relatively favorable safety profile. Based on this, a large number of classic prescriptions (14, 15) and modern compound drug preparations have been widely used in clinical practice, accumulating rich empirical evidence in improving nerve conduction velocity, alleviating pain and numbness symptoms, and promoting nerve repair. Modern pharmacological research has gradually revealed that core Chinese herbs and their active components, such as *Astragali Radix*, *Salviae Miltiorrhizae Radix et Rhizoma*, *Chuanxiong Rhizoma*, *Rhei Radix et Rhizoma*, *Silybi Herba*, and Curcumin, may exert neuroprotective effects by regulating oxidative stress, inhibiting inflammatory responses, improving microcirculation disorders, promoting the expression of neurotrophic factors, and regulating glucose and lipid metabolism disorders (16), providing scientific evidence for the intervention of TCM in DN. Beyond traditional Chinese herbal formulas, other botanical and dietary interventions have shown promise for DN. Evening primrose oil, rich in the omega-6 fatty acid gamma-linolenic acid (GLA), and the potent antioxidant alpha-lipoic acid (ALA) may also confer therapeutic benefits. The primary mechanisms involve counteracting distinct pathogenic pathways: GLA bypasses impaired Δ-6-desaturase activity in diabetes to restore the balance of vasoactive and pro-resolving lipid mediators, thereby improving nerve blood flow and reducing neuroinflammation. Concurrently, ALA directly scavenges reactive oxygen species (ROS), recycles endogenous antioxidants, and improves mitochondrial bioenergetics, thereby alleviating oxidative stress—a core driver of nerve damage (17). Furthermore, the protective role of curcumin in DN is supported by a growing body of evidence, positioning this natural polyphenol as a promising multi-target therapeutic agent. Its efficacy stems from the ability to simultaneously address several core pathological processes of DN, including chronic inflammation, oxidative stress, fibrosis and so on (18).

The global burden of DN has driven a marked expansion of research into botanical interventions. This literature is vast and heterogeneous, spanning clinical trials of complex formulae and mechanistic studies of single compounds. How can we synthesize these disparate findings and distinguish central research trends from isolated findings? Traditional review methods, though valuable, may struggle to objectively map this knowledge landscape. This raises a key question: is there a more systematic approach to navigate the field? Bibliometrics meets this need by providing a quantitative, data-driven framework to analyze the research corpus, identify core knowledge domains, and illuminate the collaborative networks and emerging trends that shape the future of botanical interventions for DN. Unlike traditional reviews, bibliometrics uses mathematical and statistical methods and employs bibliometric analysis and visualization tools such as CiteSpace and VOSviewer to conduct large-scale quantitative analysis of literature and its characteristic attributes (such as authors, institutions, countries, journals, keywords, citations, etc.) on a massive scale (19, 20). It can reveal the development history, research status, cooperation networks, knowledge base, emerging themes, and future directions of a specific discipline (21, 22). Integrating bibliometrics into the research of TCM for the prevention and treatment of DN helps to transcend the limitations of individual studies, gain a macroscopic perspective on the discipline, accurately identify key researchers and critical literature, depict the dynamic evolution of the disciplinary knowledge structure, and capture research frontiers and potential breakthroughs, following the preliminary guidelines of the literature review report on biomedical literature bibliometrics (23).

At present, there is a dearth of systematic bibliometric analyses regarding the research on botanical interventions for the improvement of DN. Therefore, this study aims to systematically utilize bibliometric methods to conduct a comprehensive analysis of research literature on the treatment of DN with botanical interventions, as indexed in authoritative databases such as the Web of Science Core Collection (WoSCC), PubMed database (24, 25). This effort is intended to bridge the gap in prior research and provide guidance for future research.

## 2 Methods and materials

### 2.1 Data sources and search strategy

**FIGURE 1:**
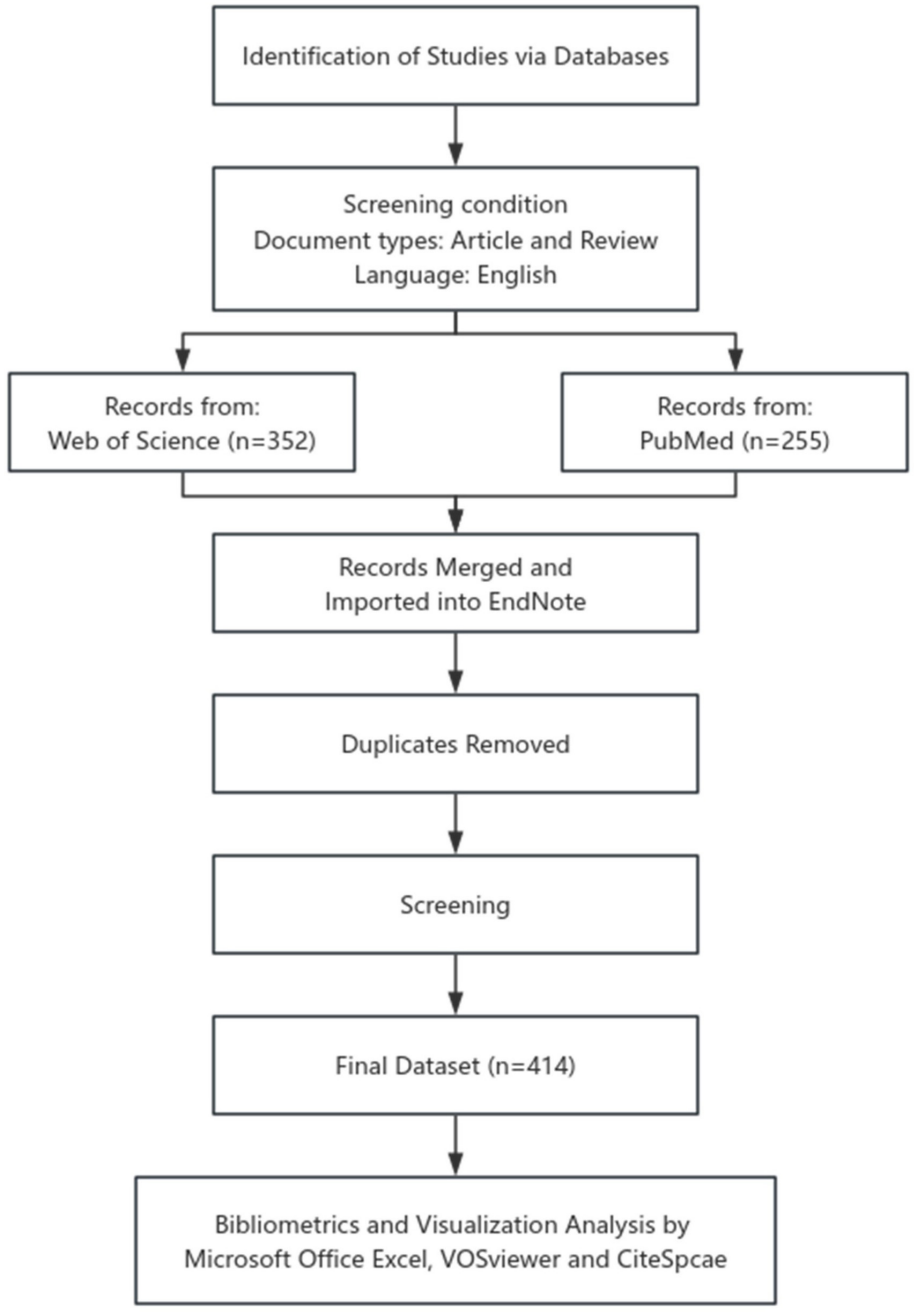
Flow chart illustrating the bibliometric analysis process.

The data used in this study were retrieved and downloaded from WoSCC (26) and PubMed (27) on July 26, 2025. The retrieval strategy in WoSCC was as follows: TS = (“Diabetic Peripheral Neuropath*” OR DPN OR “Distal Symmetric Polyneuropath*” OR Diabetic Neuropath*) AND TS = (“Traditional Chinese Medicine” OR TCM OR “herbal medicine” OR herb* OR phytotherap* OR “Plant Extracts” OR “Natural Product*” OR “natural compound*”). The retrieval strategy in PubMed was {(“Diabetic Peripheral Neuropath*”[Title/Abstract]) OR (DPN[Title/Abstract]) OR (“Distal Symmetric Polyneuropath*”[Title/Abstract]) OR (“Diabetic Neuropath*”[Title/Abstract])} AND {(“Traditional Chinese Medicine”[Title/Abstract]) OR (TCM[Title/Abstract]) OR (“herbal medicine”[Title/Abstract]) OR (herb*[Title/Abstract]) OR (phytotherap*[Title/Abstract]) OR (“Plant Extracts”[Title/Abstract]) OR (“Natural Product*”[Title/Abstract]) OR (“natural compound*”[Title/Abstract])}. The time span for the search was set from 2005-01-01 to 2025-07-25 (28). A total of 607 records were initially identified, with 352 records in WoSCC and 255 records in PubMed. To ensure high-quality content, the study included only published articles and reviews written in English, while excluding case reports, abstracts, retracted articles, meeting reports and non-English articles in the two databases (29, 30). The retrieved records from two databases were imported into the reference management software, EndNote X9 (31). Following this, the “*Find Duplicates*” function in References was employed to identify and delete redundant entries based on DOI. The detailed screening process was illustrated in Figure 1. Ultimately, a total of 414 articles were obtained as the final dataset and exported in the form of “full record and cited references” for further analysis (32).

In this search strategy, the year 2005 was selected as the starting point to capture the modern period of increasing research output on botanical interventions for DN, coinciding with the maturation of electronic database indexing and a rise in related publications. The restriction to English-language publications was consistent with prevailing bibliometric practices for mapping global research landscapes, as English serves as the predominant lingua franca of international scientific discourse. Limiting the scope to English ensured data homogeneity and enhanced the reliability of citation tracking and keyword analysis, though we acknowledge this may underrepresent contributions from non-English-speaking regions.

The screening of titles and abstracts was conducted by one reviewer, as the objective criteria (document type, topic relevance) made this efficient and sufficient for this bibliometric study. Uncertain cases were resolved by team discussion. Supplementary Data Sheet 1 provides the raw dataset, as well as the merged and cleaned dataset used for the bibliometric analysis. This supplementary file is referred to for readers who seek more granular data regarding the bibliometric analysis.

### 2.2 Bibliometric analysis and visualization

Data organization and preliminary analysis was completed in Microsoft Excel 2021 (33, 34). For the bibliometric analysis, this study utilized advanced data visualization and analysis tools (35), including VOSviewer (version 1.6.10) (36), CiteSpace (6.4.R1) (19) and bibliometrix R package (version 4.3.0) (37).

In the VOSviewer analysis, the full counting method was applied for visualizations including co-authorship networks by country and institution, co-citation analysis, and keyword co-occurrence networks (38). For the key bibliometric networks, the node size is proportional to the publication volume and line thickness represents the strength of association (39). The clustering analysis in VOSviewer employed the default VOS clustering algorithm. A minimum number of occurrences or citations threshold of 5 was typically applied for an item to be included in the network, which was adjusted based on the specific network characteristics to ensure a clear and interpretable visualization. The association strength normalization method was used to calculate the link strengths. Additionally, the layout of the network was optimized using the attraction and repulsion parameters. These indicators helped understand the cooperation patterns, intensity and core participants among researchers, institutions and countries within the field. These results also provided a comprehensive and in-depth perspective for identifying the hot topic and research trend in this field.

Trend analysis and burst detection was examined by CiteSpace through Kleinberg’s burst detection algorithm to identify keywords that increased suddenly and significantly in a specific time period (40). The time slicing length was set to one year to ensure the result sensitive and accurate. These burst words represented sudden shifts in research interests or the emergence of new hotspots, which indicated the research direction. Bibliometrix is an open-source, comprehensive bibliometric analysis software package based on the R language, which offers a complete workflow from data retrieval, cleaning to analysis and visualization (41). This package provides tools and functions to analyze and visualize bibliographic data, such as collaboration networks, keyword co-occurrences, thematic mapping and citations (42).

For the analysis of international collaborations and country/institution productivity, only the WoSCC subset (*n* = 352) was used, as WoSCC provides standardized affiliation fields that facilitate accurate extraction of geographic and institutional data. PubMed records, which often contain free-text affiliation strings, were excluded from this specific analysis to minimize misclassification. However, all PubMed records were retained for other analyses (e.g., keyword trends), and duplicate records between databases were removed based on DOI using EndNote X9 and CiteSpace to avoid double counting.

## 3 Results

### 3.1 Publication trends

**FIGURE 2:**
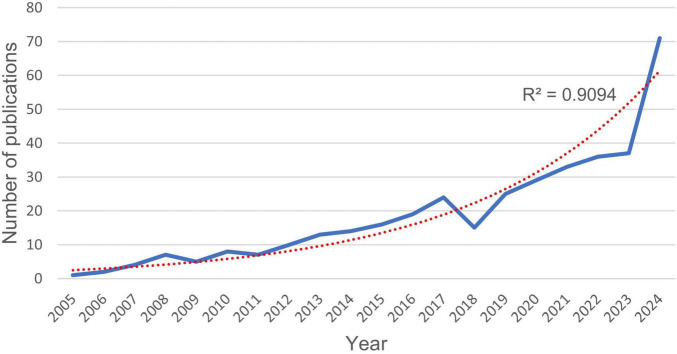
Annual publication trends in the area of the botanical interventions for DN, 2005–2024.

Annual publication output on botanical interventions for DN between 2005 and 2024 came from the merged and deduplicated dataset of WoSCC and PubMed (Figure 2). This output shows how research interest in this area has changed over the 20 years, which reveals a pronounced non-linear growth trend. The exponential model demonstrated a superior fit (R^2^ = 0.9094) compared to the linear model (R^2^ = 0.7777), so the first one works better for showing this fast-growing trend. The analysis reveals a pronounced growth trend, which can be divided into several phases. The first phase (2005–2011) was characterized by low and stable annual publication outputs, with fewer than ten articles published each year. Accelerated growth occurred from 2012 to 2017, peaking at 27 publications in 2017. After a brief decline in 2018–2019, output surged sharply from 2020 onward, reaching 71 publications in 2024. Based on this trajectory, research output is expected to continue its upward trend in 2025.

### 3.2 Geographical and institutional analysis

**FIGURE 3:**
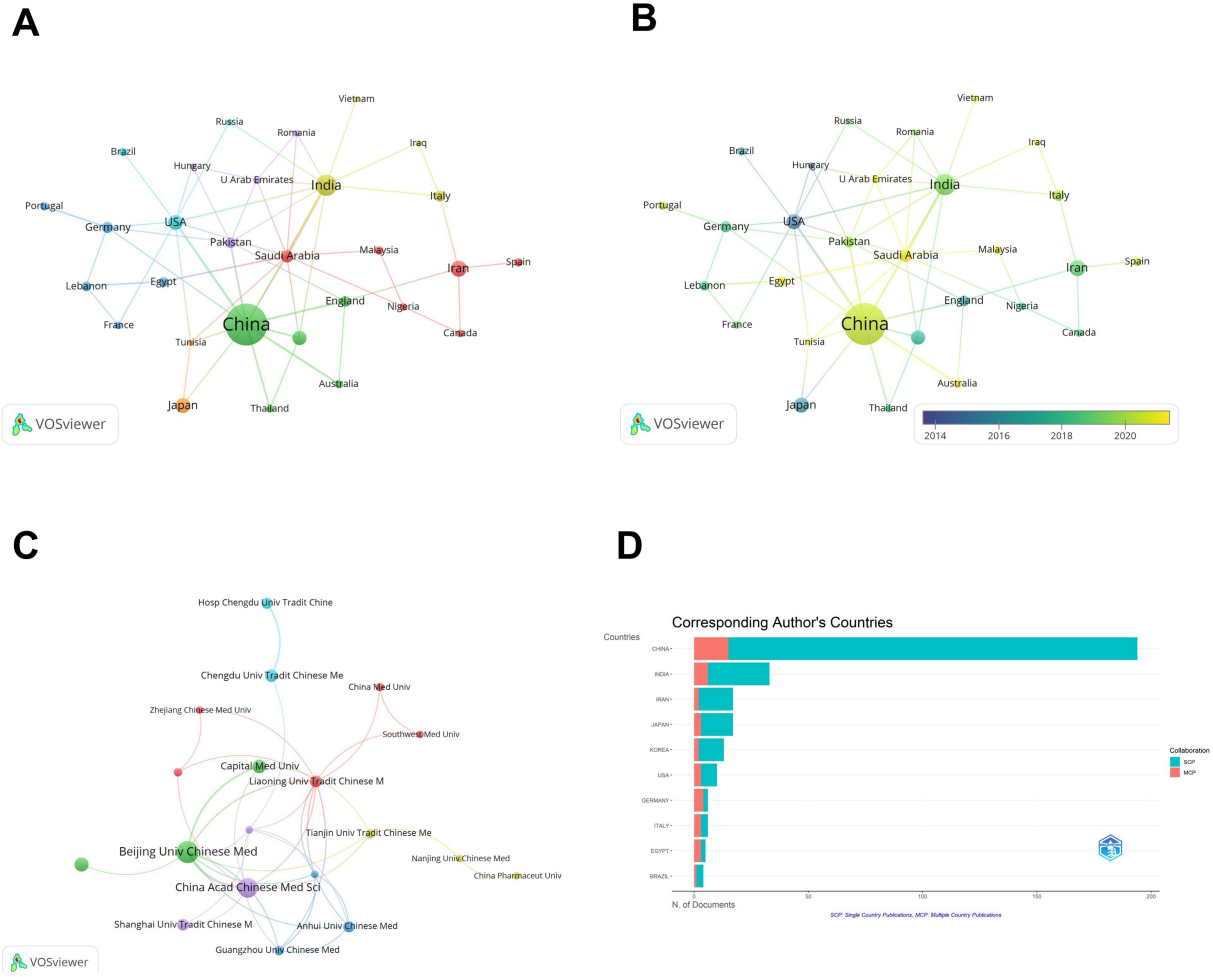
Network visualization of publications in botanical interventions for DN from 2005 to 2025. **(A)** Cooperation network of countries/regions. The node size represents the number of publications by each country, and edge thickness indicates the strength of co-authorship collaborations. **(B)** Countries with publication year; the colors of nodes and links indicate the average appearing year. **(C)** Cooperation network of research institutions, where the node size is proportional to publication volume and the edge thickness represents collaboration strength. **(D)** Visualization of corresponding author’s countries.

Global research output was overwhelmingly dominated by China (43), followed by India (Figure 3A). National collaboration analysis revealed seven clusters: Cluster 1 (Canada, Iran, Malaysia, Nigeria, Saudi Arabia, Spain); Cluster 2 (Australia, China, England, South Korea, Thailand); Cluster 3 (Egypt, France, Germany, Lebanon, Portugal); Cluster 4 (India, Iraq, Italy, Vietnam); Cluster 5 (Hungary, Pakistan, Romania, United Arab Emirates); Cluster 6 (Brazil, Russia, USA); and Cluster 7 (Japan, Tunisia). China served as a central hub with strong intra-cluster ties within Cluster 2 and trans-cluster links to India (Cluster 4) and the USA (Cluster 6). The top 10 corresponding author’s countries were shown in Table 1. Despite leading in output, China’s international co-authorship rate [multiple country publications (MCP): 7.7%] lagged behind that of the USA (30.0%) and Germany (66.7%), which had fewer total publications (Figure 3D). Institutionally, collaboration concentrated heavily within Chinese academia (Figure 3C), with Beijing University of Chinese Medicine showing the highest link strength, primarily with domestic partners like China Academy of Chinese Medical Sciences. The network exhibited minimal international integration, with non-Chinese institutions constituting less than 5% of collaboration links. This domestic focus was reflected in the high single country publications (SCP) ratios for China (SCP/Articles: 179/194), India (27/33), and Iran (15/17). Emerging contributors since 2015 included Vietnam and Saudi Arabia, the latter relying entirely on international collaboration (3 publications, MCP%: 100%), though its influence remains limited (Figure 3B).

**TABLE 1:** The top 10 corresponding author’s countries.

Country	Articles	Articles %	SCP	MCP	MCP %
China	194	55.1	179	15	7.7
India	33	9.4	27	6	18.2
Iran	17	4.8	15	2	11.8
Japan	17	4.8	14	3	17.6
Korea	13	3.7	11	2	15.4
USA	10	2.8	7	3	30.0
German	6	1.7	2	4	66.7
Italy	6	1.7	3	3	50.0
Egypt	5	1.4	2	3	60.0
Brazil	4	1.1	3	1	25.0

### 3.3 Journal analysis

**FIGURE 4:**
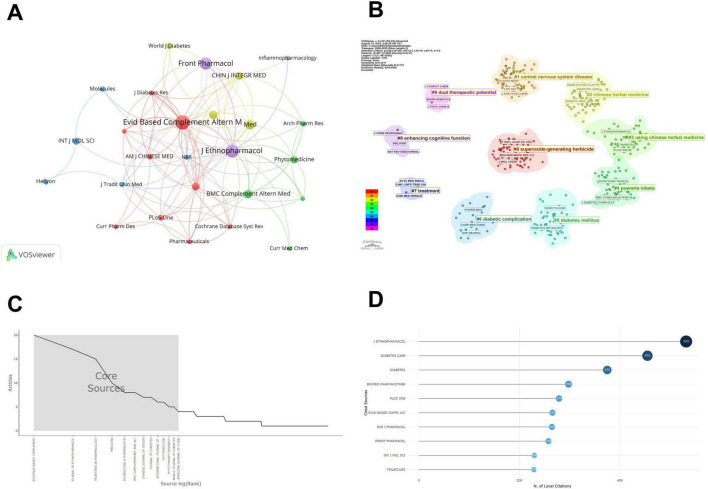
Journal citations visualization in botanical interventions for DN from 2005 to 2025. **(A)** Visualization of cited journal clusters, where node size represents the publication volume of each journal, and the edge thickness indicates the strength of collaboration between journals. **(B)** Visualization of cited journal clusters, where each color represents a distinct cluster. **(C)** Bradford’s distribution plot, illustrating the “core zone” of journals. **(D)** Top 10 cited sources in the field of botanical interventions for DN.

VOSviewer analysis grouped 26 highly cited journals into five clusters (Figure 4A): clinical medicine (Evidence-Based Complementary and Alternative Medicine), pharmaceutical chemistry (Molecules), molecular science (Oxidative Medicine and Cellular Longevity), translational medicine (Biomedicine & Pharmacotherapy), and ethnopharmacology (Journal of Ethnopharmacology). Journal of Ethnopharmacology had the highest citation links (444 citations). Biomedicine & Pharmacotherapy achieved more citations per article despite fewer publications. Specialized journals like Oxidative Medicine and Neural Regeneration Research each received 187 citations.

The Bradford distribution plot (Figure 4C) highlights the “core zone” of journals, which includes the Journal of Ethnopharmacology, Evidence-Based Complementary and Alternative Medicine, and Frontiers in Pharmacology. These journals are the most productive in terms of publications in the field of botanical interventions for diabetic neuropathy, with a significant concentration of publications. Table 2 provides citation metrics for these core journals, including the H-index, G-index, total citations (TC), and the number of publications (NP). For example, the Journal of Ethnopharmacology has the highest H-index of 12 and the largest total citation count (444). These citation metrics underline the significant impact of these core journals in shaping the research landscape.

**TABLE 2:** The sources’ impact.

Source	H_index	G_index	TC	NP
Journal of Ethnopharmacology	12	17	444	17
Evidence-Based Complementary and Alternative Medicine	9	14	205	20
Frontiers in Pharmacology	9	14	208	15
Biomedicine & Pharmacotherapy	7	8	460	8
BMC Complementary and Alternative Medicine	7	8	142	8
Chinese Journal of Integrative Medicine	5	7	122	7
International Journal of Molecular Sciences	5	6	184	6
Journal of Diabetes	5	7	174	7
Phytomedicine	5	6	200	6
American Journal of Chinese Medicine	4	4	308	4

Figures 4C, D and Table 2 further indicate a split between high-output journals with lower citations (such as Evidence-Based Complementary and Alternative Medicine) and those with moderate output but higher citation impact (such as Journal of Ethnopharmacology and Biomedicine & Pharmacotherapy) (44). Network analysis highlighted Frontiers in Pharmacology connecting clinical and mechanistic clusters. CiteSpace confirmed Chinese herbal (Cluster 2) medicine and nerve repair (Cluster 1) as core cluster (Figure 4B).

### 3.4 Keyword analysis

#### 3.4.1 Keyword cluster analysis

In this study, keywords were extracted from all documents and analyzed by VOSviewer (45). A total of 43 keywords were selected for visualization. The top 15 high-frequency keywords were shown in Table 3. The most frequently appearing keyword was “diabetic peripheral neuropathy” (Total link strength: 309), followed by “oxidative stress” (255) and “traditional Chinese medicine” (142) (46).

**TABLE 3:** The top 15 high-frequency keywords.

Rank	Keyword	Occurrences	Total link strength
1	Diabetic peripheral neuropathy	126	309
2	Oxidative stress	76	255
3	Traditional Chinese medicine	45	142
4	Diabetic neuropathy	44	105
5	Diabetes mellitus	40	109
6	Peripheral neuropathy	38	112
7	Rats	38	95
8	Mechanisms	30	136
9	Apoptosis	29	117
10	Plant sciences	25	85
11	Herbal medicine	24	78
12	Network pharmacology	24	64
13	Pain	24	92
14	Expression	22	82
15	Neuropathic pain	23	66

**FIGURE 5:**
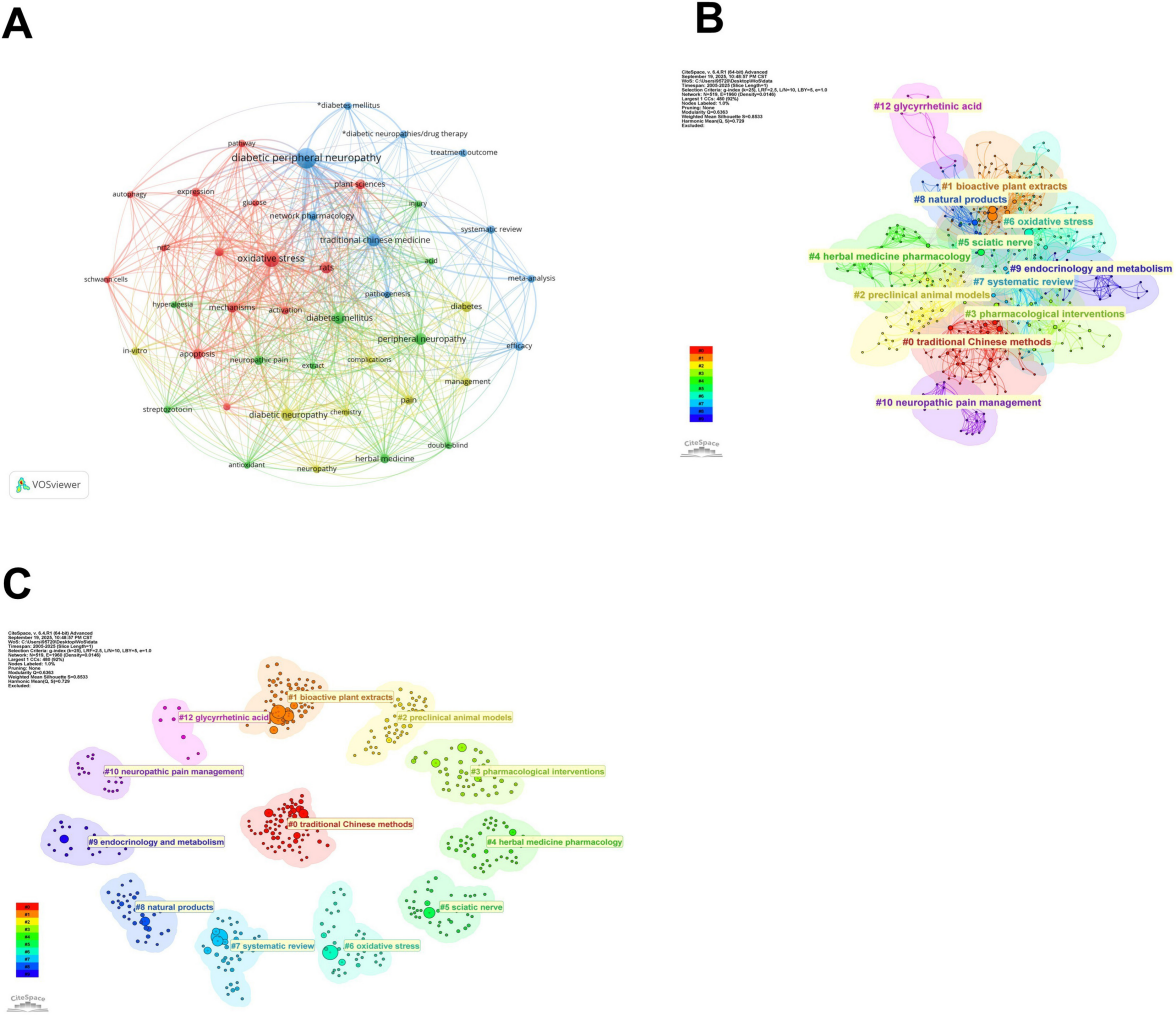
Visualization map of keywords. **(A)** Network visualization of the keywords co-occurrence analysis, where node size corresponds to keyword frequency, and edge thickness reflects the strength of co-occurrence relationships. **(B)** Cluster analysis of high-frequency keywords, where colors indicate different clusters. **(C)** Keyword co-occurrence network with a circular layout.

The keywords were divided into four clusters with different color in Figure 5A. (1) Molecular mechanisms and experimental research (red dots), which contained 14 keywords, including “oxidative stress,” “activation,” and “mechanisms.” (2) Herbal medicine and experimental treatments (green dots), there were 11 keywords, including “diabetes mellitus,” “herbal medicine,” and “peripheral neuropathy.” (3) Research methods and models (blue dots), which contained 10 keywords, including “meta-analysis,” “systematic review,” and “network pharmacology.” (4) Complication management and *in vitro* research, there were 8 keywords, including “complications,” “*in vitro*,” and “management” (47). CiteSpace was also used to perform cluster analysis; 12 clusters were generated: #0 traditional Chinese methods #1 bioactive plant extracts #2 preclinical animal models #3 pharmacological interventions #4 herbal medicine pharmacology #5 sciatic nerve #6 oxidative stress #7 systematic review #8 natural products #9 endocrinology and metabolism #10 neuropathic pain management and #12 glycyrrhetinic acid (48–50). The interrelationships and connection strengths among clusters were depicted in Figure 5B, while the core of the studies and the dispersion degree of key words were illustrated in Figure 5C.

#### 3.4.2 Future trends

**FIGURE 6:**
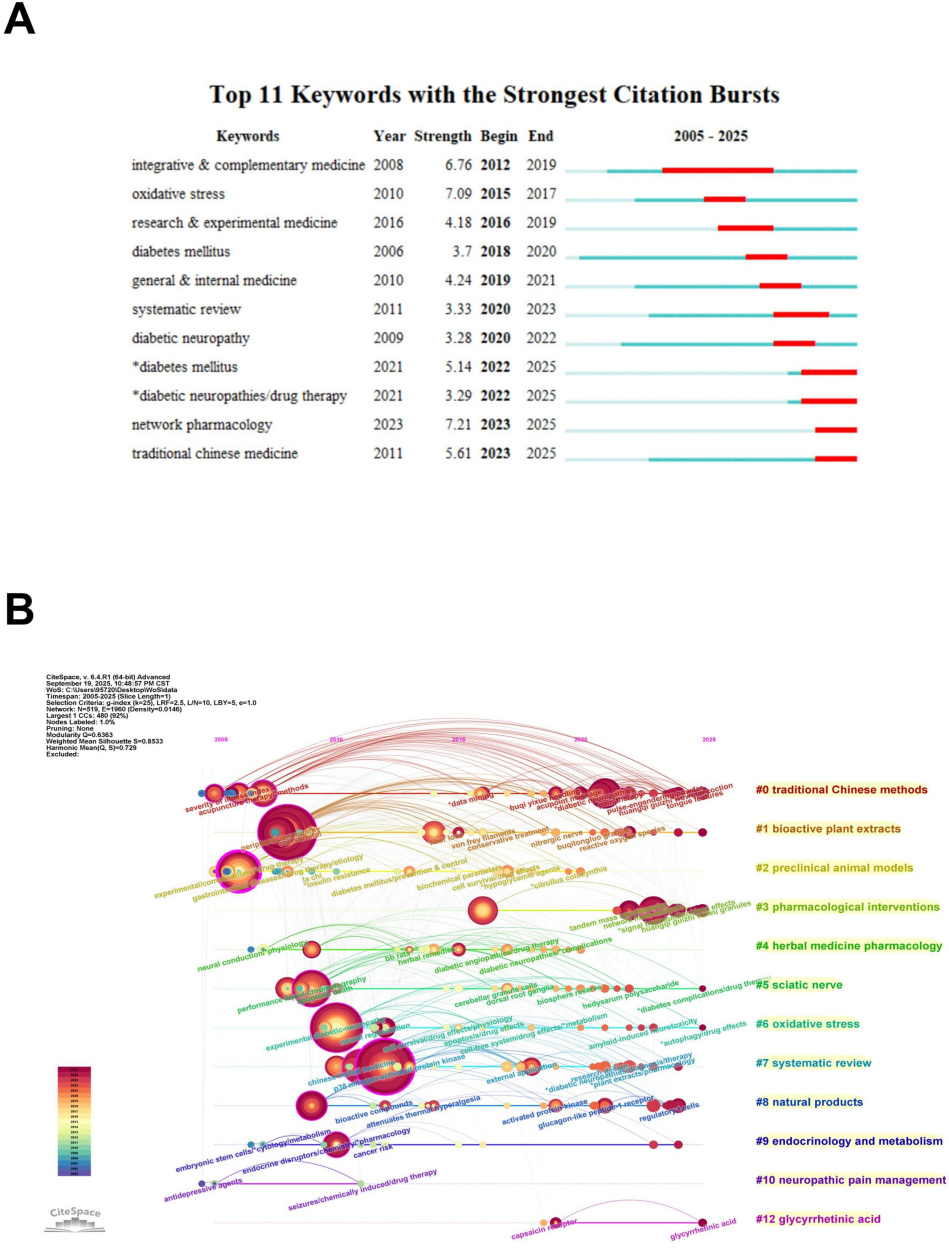
Visualization in botanical interventions for DN from 2005 to 2025. **(A)** Cluster analysis of top 11 keywords with strongest citation bursts. **(B)** The timeline chart of keywords in the area of botanical interventions for DN, showing how key research themes have evolved over time.

Keyword burst detection was performed using CiteSpace to identify research frontiers, and the results are presented in Figure 6A (51). In the figure, the blue line represents the timeline from 2005 to mid-2025, and the red segments indicate the duration of a keyword’s burst of activity. The top 11 keywords with the strongest citation bursts are listed. The keyword with the greatest burst strength was “network pharmacology” (Strength = 7.21) (52). This was followed by “oxidative stress” (Strength = 7.09), which showed a concentrated burst from 2015 to 2017 (53). Several keywords burst recently and are still active, indicating current research fronts. These include “network pharmacology” (2023–2025) and “traditional Chinese medicine” (2023–2025). The timeline view of keyword clusters (Figure 6B) also shows how the field has changed over time.

### 3.5 Nutritional relevance of bibliometric findings

The keyword co-occurrence analysis (Figure 5A) highlights key terms such as “oxidative stress” and “Nrf2” as central to the network, with “traditional Chinese medicine” also frequently mentioned. Burst detection analysis (Figure 6A) identifies “network pharmacology” as a rapidly emerging topic with a strong citation burst from 2023 to 2025. Journal co-citation patterns (Figure 4A) show that journals in oxidative medicine and ethnopharmacology, such as Oxidative Medicine and Cellular Longevity and Journal of Ethnopharmacology, receive significant citations, reflecting distinct research directions. Keyword clustering (Figure 5A) identifies three key thematic areas with nutritional relevance: oxidative stress mechanisms, herbal medicine, and natural products research. These findings highlight the continued focus on plant-based compounds and their biological mechanisms, supporting their potential nutritional applications.

## 4 Discussion

### 4.1 Hotspots and frontiers

This bibliometric analysis delineated the evolving research landscape of botanical interventions for DN over the past two decades. Publication trends, which were non-linear, demarcated three distinct phases: initial development (2005–2011), accelerated growth (2012–2017), and a sharp surge post-2020, reflecting rapidly expanding activity. Geographically, China dominated the research output, yet the collaboration network revealed a pattern of persistent regionalization within a globalizing field. Specifically, China’s international co-authorship rate remained substantially lower than that of the USA and Germany, pointing to a strong domestic focus and limited global integration at the institutional level. This pattern of regionalized academic activity is reflected in the field’s distinct communication channels, as revealed by journal co-citation analysis. Journal co-citation analysis further showed a divergence between high-output clinical journals and traditional medicine journals, which drove citation impact. Specialized journals focusing on mechanisms like oxidative stress exerted notable influence, often acting as interdisciplinary hubs. Finally, keyword analysis identified the application of advanced methodologies such as network pharmacology to traditional medicine formulas as the current research frontier. Collectively, these findings portrayed a dynamic and maturing field, characterized by concentrated productivity, distinct communication patterns, and evolving research fronts. Consequently, we identified two major research focuses concerning natural products in DN. The first frontier pertained to the role of natural products in DN, specifically regarding trophic regulation and adjunctive intervention. The second theme focused on the relationship between signaling pathways and the regulation of DN.

The findings of the bibliometric analysis in this study only depicted the features of the global academic publishing landscape and did not comprehensively mirror the actual prevalence of DN in various regions. This limitation was primarily attributed to factors like uneven research resource allocation and language obstacles. In low- and middle-income nations, DN cases might have encountered disparities in diagnostic criteria, limited screening reach, and inadequate diagnostic and therapeutic facilities. These influential elements were not entirely captured in the existing data and warranted more in-depth examination in forthcoming studies with expanded datasets.

#### 4.1.1 Nutritional regulation and adjunctive intervention of natural products in the field of DN

Various intervention strategies were available for DN, including glycemic control (e.g., metformin), enhancement of microcirculation (e.g., prostaglandin injection), nerve regeneration (e.g., methylcobalamin), and pain relief (e.g., pregabalin) (54–56). Although these drugs improved the condition to a certain extent, their clinical application exhibited significant limitations. Consequently, novel intervention approaches that are both efficacious and safe are urgently required in this field. Natural products, known for their diverse types, gentle medicinal qualities, and ability to target multiple pathways, have garnered significant attention from researchers and emerged as a focal point in nutritional modulation and adjunctive interventions for DN (56, 57).

A series of chain reactions involving metabolic disorders, inflammatory responses, and oxidative stress, triggered by hyperglycemia, constituted the core mechanisms that led to severe microvascular and neurological damage. The potential value of natural products resided in their capacity to mitigate these damaging effects through trophic regulation. Regarding oxidative stress, several studies demonstrated the modulatory role of natural products in countering this condition. A preclinical study revealed abnormal nociception in a mouse model of streptozotocin-induced diabetes following intervention with 7-hydroxy-3,4-dihydropalatine. The investigators observed that the mice exhibited reduced plasma malondialdehyde (MDA) concentrations, which correlated with the activation of 5-hydroxytryptamine (5-HT) receptors and enhanced expression of antioxidant enzymes (58). In another animal experiment utilizing the same mouse model, researchers found that mice intervened with the natural polyphenolic compound resveratrol displayed increased thermal pain thresholds and mechanical pain sensitivity, potentially linked to the scavenging of ROS and the inhibition of lipid peroxidation (59). Furthermore, some studies indicated that natural products such as berberine and curcumin demonstrated potential activity in inhibiting oxidative stress in *in vitro* experiments or animal models. However, most of these studies remained in the preliminary stage, with the evidence level confined to preclinical findings, indicating that the field required further exploration (60, 61).

The regulatory functions of natural products in inhibiting inflammatory responses and maintaining neurotransmitter homeostasis received considerable attention. Some studies demonstrated that *Schefflera arboricola* (SA) tablets significantly reduced serum concentrations of pro-inflammatory factors, including IL-1β and TNF-α, in diabetic model rats, potentially due to the inhibition of the NF-κB pathway (62). In another animal experiment involving a diabetic rat model, the researchers observed that the TNF-α content in the sciatic nerve of rats following chlorogenic acid intervention was significantly reduced, and the cellular inflammatory damage was less severe. This finding may be associated with the modulatory effect of chlorogenic acid on inflammatory factors (63). However, it is important to note that these data derived solely from animal experiments, and there existed a substantial disparity in pathological characteristics when compared to humans, necessitating verification through subsequent clinical studies.

The research value of natural products extended beyond the domain of DN, with several studies indicating their potential regulatory role in various neurological disorders. For Alzheimer’s disease, researchers discovered that resveratrol inhibited the polymerization of β-amyloid (Aβ) and promoted the degradation of Aβ by proteases in primary hippocampal neurons derived from rats. For Parkinson’s disease, resveratrol activated the anti-apoptotic factor Bcl-2 and inhibited caspase-3 activity in SH-SY5Y neuroblastoma cells. This effect appeared to correlate with the attenuation of dopamine oxidation-induced neurotoxicity (59). In the realm of TCM research, Mudan Granules in combination with mecobalamin and *Cassiae Semen* exhibited potential nutritional modifying effects in the intervention of DN (64, 65). The integration of TCM physical therapy, such as acupuncture, with herbal injections appeared to enhance the efficacy of DN treatment (66, 67). Nonetheless, these studies possessed small sample sizes and low levels of evidence; thus, further high-quality clinical studies were necessary to validate the pertinent conclusions.

Despite the numerous potential modulation modalities of natural products in the field of DN, it faced significant challenges in transitioning from basic research to clinical application. Firstly, the active components and specific mechanisms of action of most natural products remained unclear, with some studies limited to the surface level of extracts. For instance, the target of action for SA tablets had not been identified, systematic safety evaluation data were lacking, and the range of clinically applicable doses had not been defined (62). Secondly, the pharmacokinetics of natural products were complex, with low bioavailability, making it difficult to maintain effective therapeutic concentrations at the lesion site. For example, the oral bioavailability of resveratrol was only approximately 2%. The development of nanocarriers to enhance cell targeting represented a primary direction for future research (59, 68, 69). Thirdly, the induced animal models of diabetes failed to fully simulate the process of DN lesions. Significant differences existed between the results of animal experiments and clinical trials, necessitating ongoing optimization of the models and experiments.

#### 4.1.2 The regulatory relationship between signaling pathways and DN

In the intricate pathological progression of DN, numerous signaling pathways collaboratively regulated functions. Oxidative stress and inflammatory responses, the fundamental pathogenesis of DN, garnered consistent interest from researchers. Recent studies have found that through the interaction of signaling pathways, the dynamic fluctuations of lipid metabolism and the abnormal transmission of exosomes between cells can further exacerbate the development of DN. This perspective offered a novel angle for exploring the mechanism of DN.

Among numerous signaling pathways, the Nrf2 pathway played a pivotal role. The Nrf2 factor served as a crucial transcription factor that enabled cells to combat oxidative stress. It counteracted oxidative stress responses by regulating downstream genes (60, 70). Under hyperglycemic conditions, the accumulation of advanced glycation end products (AGEs) and the activation of protein kinase C (PKC) promoted the development of oxidative stress. In an animal experiment involving a diabetic model mouse, researchers observed that the expression of Nrf2 in the sciatic nerve of mice increased, while the concentration of MDA decreased following intervention with SA. This change potentially correlated with the improvement of the pain threshold in the mice (62).

In addition, Nrf2 factor may exhibit a cross-regulatory effect in the inhibition of inflammatory responses. NF-κB factor served as a crucial transcription factor that mediated these inflammatory responses. Nrf2 inhibited the activity of the NF-κB pathway by competing for transcriptional binding targets or by inducing the production of carbon monoxide, which suppressed the malignant development of inflammatory responses. In animal experiments employing the same model, the content of TNF-α in mice decreased, while the expression of TGF-β increased. This phenomenon might have related to the indirect inhibition of the NF-κB pathway by bergenin (70, 71). However, these findings were confined to a single animal model and cannot be directly extrapolated to their effects in humans.

It was noteworthy that, in addition to hyperglycemia, disorders in lipid metabolism also activated the signaling pathways of inflammatory responses, and promoted the pathological development of DN. The study conducted by Karimi et al. (72) involving 144,226 diabetic patients confirmed, to some extent, the potential connection between lipid variability and microvascular complications. The results indicated that for every 1-unit increase in the variability of low-density lipoprotein (LDL), high-density lipoprotein (HDL), and triglycerides (TG), the risk of microvascular complications increased by 11, 9, and 8%, respectively (72). This phenomenon may have been related to the activation of pathways such as MAPK and the antagonism of the Nrf2 pathway. Exosomes, which acted as mediators of intercellular communication, also emerged as detrimental factors in neuroinflammation. Studies demonstrated that in environments characterized by lipid variation, the contents of exosomes released by Schwann cells and other sources changed. These abnormal exosomes activated the STAT3 pathway, hindered autophagy in Schwann cells, and inhibited neural repair (73). Collectively, these studies provided support for the hypothesis that apolipoprotein E (APOE)-driven lipid imbalance and fluctuations synergistically aggravated the progression of neuropathy.

The PI3K/Akt pathway also played a significant role in the development of DN (74). In a hyperglycemic environment, the activity of this pathway became inhibited, resulting in an increase in nerve cell apoptosis. In an *in vitro* experiment involving rat Schwann cells, researchers observed that the apoptosis rate of cells intervened with artesunate was lower than that of the control group. This finding may be related to artesunate’s ability to activate the PI3K/AKT pathway, suppress pro-apoptotic factors such as caspase-3, and upregulate anti-apoptotic proteins such as Bcl-xL (75).

Furthermore, the AMPK pathway, which senses the energy state of cells, and the MAPK pathway, which mediates inflammatory responses, may also have potential connections to the progression of DN (76). However, existing research on signaling pathways of DN exhibited notable limitations. Firstly, most studies concentrated solely on the independent roles of individual pathways, and lacked systematic analyses of the interactions between these pathways. Additionally, most experiments relied on *in vitro* cells or animal models, resulting in a relative scarcity of clinical evidence.

### 4.2 Limitations

This study provides a comprehensive overview of the research landscape of DN plant-based interventions through a bibliometric analysis. However, several limitations should be acknowledged. First, this study only included English articles and reviews published in the past 20 years from the WoSCC and PubMed databases, excluding non-English publications, case reports, conference abstracts, etc. This may have led to the omission of some regional research findings, particularly resulting in insufficient coverage of traditional medicine studies from non-English-speaking countries. Second, the data for 2025 only covers publications up to July, failing to include the full year’s literature, which might affect the accuracy of publication trends for that year. Third, potential errors might exist in the processes of author name disambiguation and institutional name standardization. Furthermore, the citation lag effect could interfere with the analysis of the impact of literature published in 2024–2025. Additionally, the interpretation of geographical research output is based on absolute publication counts. While this effectively reflects the scale and volume of different national research ecosystems, it may not directly indicate relative research efficiency or specialization, as the data were not normalized by metrics such as per-capita GDP or research population size. Nevertheless, given that English is one of the most widely used scientific languages, this study still encompasses a substantial volume of literature in this field. Therefore, despite the aforementioned limitations, the conclusions drawn from this study remain credible.

### 4.3 Future directions

#### 4.3.1 Deepening mechanistic and multi-target research

Future research needs to integrate molecular biology and multi-omics technologies to deeply elucidate the specific molecular mechanisms by which core active ingredients (e.g., curcumin, astragalus polysaccharides) regulate key signaling pathways such as NF-κB, AMPK, and Nrf2, which may help clarify their precise targets in neural repair (77). Experimental validation of network pharmacology findings should be strengthened. Combined with technologies like gut microbiota sequencing and metabolomics, future directions could aim to clarify the potential synergistic mechanisms of plant interventions involving “multi-component, multi-target, multi-pathway” interactions (78). Simultaneously, attention should be paid to emerging mechanisms such as lipid fluctuations and exosome-mediated neuroinflammatory signaling, thereby expanding the depth and breadth of research, while noting that current evidence for many mechanisms is primarily derived from preclinical models.

#### 4.3.2 Shifting focus from network pharmacology to prospective clinical validation

Priority should be given to conducting large-scale, multi-center, randomized controlled trials with long-term follow-up to robustly assess the efficacy and safety of plant interventions. Standardizing the dosage, treatment duration, and efficacy evaluation systems for plant-based interventions is essential, with particular focus on long-term safety and tolerability. Establishing unified diagnostic and efficacy evaluation criteria for DN, and standardizing the operational procedures of traditional therapies such as acupuncture and herbal foot baths, are crucial to enhance the comparability and reproducibility of research findings. Concurrently, strengthening the integration of a nutritional science perspective is essential. This includes conducting human exposure dose assessments, dose-response relationship analyses, and research on the impact of food matrices on the bioavailability of plant active compounds, thereby providing more precise guidance for clinical applications.

#### 4.3.3 Strengthening international and cross-regional collaboration

Breaking down regional research barriers and establishing a global DN plant intervention research collaboration network is vital, focusing on promoting cooperation between low-output and high-output institutions and facilitating the sharing of clinical samples, data, and research platforms. Promoting the development of international standards for areas like herbal medicine fingerprinting and quantitative analysis of active ingredients could help unify research methods and data reporting norms. Furthermore, it is recommended to upload research data (including raw literature search results, analysis scripts, visualization parameters, etc.) to stable repositories like Zenodo to ensure research reproducibility and facilitate re-analysis. Additionally, encouraging cross-regional joint funding programs, focusing on under-researched areas, could help promote more balanced global research development.

## 5 Conclusion

This study maps the 20 years evolution of research on botanical interventions for DN. Our analysis reveals a field undergoing rapid transformation, characterized by three key trends: exponential growth in publications since 2020; a dominant contribution from China alongside limited international collaboration; and a thematic shift from basic oxidative stress mechanisms toward complex, multi-target approaches like network pharmacology, particularly for TCM formulas. Looking forward, the findings point to clear priorities. A pressing need is to strengthen international cooperation to validate results across diverse populations. The field must also prioritize developing standardized outcome measures and conducting rigorous, multicenter clinical trials to firmly establish the efficacy and safety of promising botanicals. Beyond clinical translation, future work should deepen mechanistic investigations and address critical safety considerations, such as potential botanical-drug interactions. Addressing these priorities will be essential for translating the potential of botanical interventions into evidence-based therapies for DN.

## Data Availability

The original contributions presented in this study are included in this article/Supplementary material, further inquiries can be directed to the corresponding author.

## Footnotes

1
https://diabetesatlas.org/resources/idf-diabetes-atlas-2025/
